# Development of a nomogram to predict in-ICU mortality of elderly patients with sepsis-associated liver injury: an analysis of the MIMIC-IV database

**DOI:** 10.3389/fmed.2025.1516853

**Published:** 2025-03-26

**Authors:** Xuemei Hu, Jianbao Wang, Susu Cao, Aolin Xia, Xiaocong Jiang, Tianfeng Hua, Min Yang

**Affiliations:** ^1^The Second Department of Intensive Care Unit, The Second Affiliated Hospital of Anhui Medical University, Hefei, Anhui, China; ^2^The Laboratory of Cardiopulmonary Resuscitation and Critical Care Medicine, The Second Affiliated Hospital of Anhui Medical University, Hefei, Anhui, China; ^3^The Department of Emergency, The Second Affiliated Hospital of Anhui Medical University, Hefei, Anhui, China

**Keywords:** sepsis-associated liver injury, in-ICU mortality, nomogram, lasso regression, MIMIC-III database, model

## Abstract

**Background:**

Sepsis-associated liver injury (SALI) is a frequent and lethal complication among critically ill patients in the intensive care unit (ICU). Despite its significance, there has been a notable lack of specialized tools for evaluating the in-ICU mortality risk in these patients. This study seeks to address this gap by developing a practical nomogram to predict risk factors associated with in-ICU mortality in patients suffering from SALI.

**Methods:**

Data were extracted from the MIMIC-IV database, a Critical Care Public Medical Information Mart. The diagnostic criteria for sepsis adhered to the Sepsis 3.0 guidelines, requiring a SOFA score of ≥ 2. SALI was defined as total bilirubin (TBIL) levels > 2 mg/dL in patients with sepsis and an International Normalized Ratio (INR) > 1.5. Lasso regression analyses were conducted on the training set (*n* = 653) to develop a predictive nomogram model. Receiver Operating Characteristic (ROC) curves were generated to evaluate model discrimination. Model calibration was assessed through calibration curves and Hosmer-Lemeshow goodness-of-fit tests. Clinical decision curves were plotted to analyze the net benefit of the model and evaluate its clinical applicability.

**Results:**

A total of 934 elderly patients with SALI were included in the study. Random seeds were allocated in a 7:3 ratio, resulting in training and validation sets comprising 653 and 281 patients, respectively. Variables were selected using lasso regression, culminating in the inclusion of six final variables within the model. The nomogram was evaluated against standard ICU scoring systems, specifically SAPS II and SOFA scores, yielding AUROC values of 0.814, 0.798, and 0.634 for the training set, respectively. Conversely, the validation set demonstrated AUROC values of 0.809, 0.791, and 0.596. The nomogram exhibited strong predictive performance for in-ICU outcomes. *P*-values from the Hosmer-Lemeshow goodness-of-fit test for both training and validation sets were recorded at 0.627 and 0.486, respectively, indicating good fit quality. Decision curve analysis revealed that the nomogram consistently provides greater net benefits compared to SAPS II and SOFA scores.

**Conclusion:**

A prediction model of in-ICU mortality in SALI elderly patients was established by screening variables through lasso regression. Nomgram was the best predictor of in-ICU mortality in SALI patients, which has a high reference value and clinical application.

## 1 Introduction

Sepsis is a life-threatening organ dysfunction caused by infection and is one of the common causes of death in patients admitted to the intensive care unit (ICU) (1). The production of endotoxins and the release of inflammatory factors in sepsis lead to an abnormal immune response that impairs the functioning of many organs, including the liver and kidneys (2). The liver plays a central role in metabolic and immune homeostasis, and liver injury is one of the common complications in patients with septic shock and one of the risk factors for death in septic patients (3, 4). Early liver damage is caused by inflammation and microcirculation disorders, which can be recovered later with treatment. The mechanism of late injury may be related to organ failure caused by sepsis (5).

Sepsis-associated liver injury (SALI) is one of the most common complications in patients with sepsis. The mechanism is that the onset of primary dysfunction is usually associated with inadequate hepatic perfusion, which leads to liver injury and further complications such as diffuse intravascular coagulation and multi-organ failure (6). Secondly, sepsis also causes dysfunction of the intestinal microcirculation, prompting leakage of intestinal toxins and bacteria into the liver through the portal vein, which can lead to hepatitis (7). There are several main types of SALI: ischemic-hypoxic liver injury, cholestatic liver injury, and hepatocellular injury (8). Ischemic-hypoxic liver injury results from hepatic hypoperfusion and hypoxemia, characterized by a significant elevation in serum aminotransferase levels, along with coagulation abnormalities and minimal jaundice (9). Cholestasis is primarily defined by hyperbilirubinemia or jaundice, which may be linked to impaired bile formation and reduced motility (10). The classification of hepatocellular injury types predominantly relies on drug-related liver injury criteria established in the literature (11). There is a lack of clarity about the mechanisms by which SALI occurs, which may be related to the following pathways, including microcirculation and endothelial damage (12), intestinal flora dysbiosis (13), inflammatory factor imbalance (7), oxidative stress (14), and mitochondrial metabolic disorders (15), among other points. Studies have shown that liver failure is an independent risk factor for multi-organ dysfunction and high mortality in patients with sepsis (16). However, there are no studies of in-ICU mortality prediction models for patients with SALI. Therefore, the purpose of this paper is to explore the independent risk factors for the development of SALI.

Sepsis is most prevalent among the elderly, infants, and individuals with certain underlying conditions that compromise the immune system. As society ages, the population of elderly patients is growing, leading to an increase in the incidence of sepsis (17). Reducing the mortality and disability rates of SALI in the elderly population is one of the current issues that needs to be addressed. Nomograms are visual statistical tools that integrate various data to develop continuous scoring systems reflecting individual risk probabilities accurately (18). Therefore, it is crucial to establish an accurate dynamic nomogram, which has important implications for both individuals and society through prognostic and clinical management decisions.

## 2 Materials and methods

### 2.1 Data source

This study used a retrospective cohort design. Data were obtained from the Medical Information Mart for Intensive Care (MIMIC)-IV database (version 3.0), which includes patients from 2020 to 2022 in addition to those from version 2.2, access to MIMIC-IV v3.0 (physionet.org). The MIMIC database is a large, freely available database that contains de-identified health-related data for over 40,000 patients admitted to the intensive care unit at Beth Israel Deaconess Medical Center in Boston, Massachusetts (19). The first author, Xuemei Hu, completed the “data or specimen research” training and was granted permission to use public data (ID: 13677449). As this study was conducted using an anonymous public database by the review board protocol, ethical consent was not required.

### 2.2 Population selection criteria

The inclusion criteria consisted of the following: (1) Meet the diagnostic criteria of sepsis 3.0 (1). (2) Patients admitted to the ICU for the first time and have been hospitalized for at least 24 h. (3) age ≥ 60 years. (4) Meet the definition of SALI: total bilirubin level > 2 mg/dL (34.2 μmol/L), international normalized ratio > 1.5 (16, 20).

The exclusion criteria encompassed: (1) does not meet the diagnostic criteria for sepsis 3.0. (2) ICU stay time less than 24 h. (3) age < 60 years. (4) did not meet the diagnostic criteria for SALI. (5) patients with previous liver disease, liver cirrhosis, and other liver disease-related diseases.

### 2.3 Data extraction and management

The study extracted relevant variables from the MIMIC-IV database: (1) patient general information; (2) past disease history; (3) severity scores, including Simplified Acute Physiology Score II (SAPS II), Sequential Organ Failure Assessment (SOFA), Systemic Inflammatory Response Syndrome (SIRS), Glasgow Coma Scale (GCS); (4) vital signs and biochemical indicators of patients admitted to ICU within 24 h; and (5) treatment after ICU admission. (6) outcome: death after ICU admission.

Variables with data missing rates greater than 30% were excluded. For the variables included in the analysis, we used R version 4.2.2 for data cleaning and input of missing values and processed missing values based on R’s “randomForest” package and used the RF method to handle missing values and the multiple imputation method to generate five input datasets for Lasso regression analysis. Specific details are provided in Supplementary Figure 1.

### 2.4 Statistical analysis

This study was analyzed and plotted through the R version 4.2.2, and the software package used is detailed in Supplementary Table 1. Categorical variables were analyzed for differences in distribution between the two groups using the chi-square test (or Fisher’s exact probability method), continuous information that conformed to a normal distribution was described in the form of (means ± standard deviation) and analyzed for differences in distribution between the two groups using the *t*-test, and information that did not conform to a normal distribution was described in the form of medians (percentiles) and analyzed for differences in distribution between the two groups using the rank-sum test. The results of intergroup comparisons were expressed as *p*-values.

A total of 70% of the patients were randomly selected as the training set, and a nomogram visualization model was developed. The predicted outcome was the patient’s risk of dying during ICU. Receiver operating characteristic (ROC), calibration curve and decision curve analysis (DCA) were constructed to further evaluate the applicability of the model.

## 3 Results

### 3.1 Patient characteristics

**FIGURE 1:**
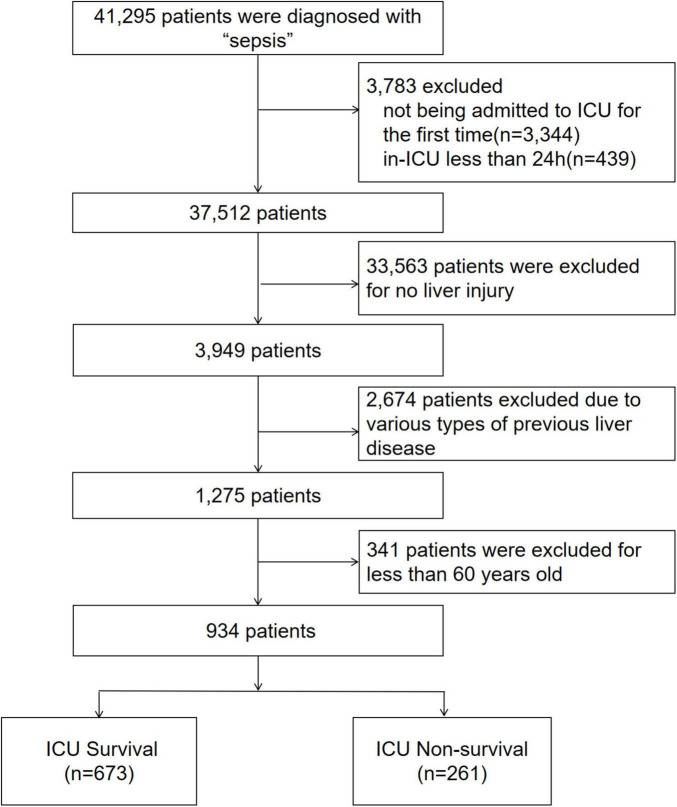
Flow chart.

A total of 934 elderly patients with SALI were enrolled in this study (Figure 1). They were categorized into survival group (*n* = 673) and non-survival group (*n* = 261) based on ICU mortality. Details of the specific subgroups and clinical baseline information are shown in Table 1.

**TABLE 1:** Patients’ baseline characteristics.

Variables	Survival (*n* = 673)	Non-survival (*n* = 261)	*P*-value
**General features**			
Gender, woman (%)	414 (61.5)	155 (59.4)	0.601
BMI (kg/m^2^)	27.40 (23.46, 31.47)	27.92 (24.22, 32.26)	0.105
Age (year)	76.12 (69.24, 83.68)	75.71 (69.21, 82.51)	0.371
**Comorbidity, *n* (%)**			
MI, *n* (%)	137 (20.4)	66 (25.3)	0.121
CPD, *n* (%)	160 (23.8)	66 (25.3)	0.69
PUD, (%)	30 (4.5)	10 (3.8)	0.807
DM, *n* (%)	194 (28.8)	65 (24.9)	0.263
RD, *n* (%)	202 (30.0)	96 (36.8)	0.056
MC *n* (%)	158 (23.5)	68 (26.1)	0.459
**Vital signs^a^**			
Heart rate (min-1)	87.37 (75.79, 99.00)	96.00 (81.86, 111.54)	< 0.001
SBP (mmHg)	109.28 (102.33, 118.91)	102.28 (94.79, 110.77)	< 0.001
DBP (mmHg)	59.73 (54.50, 66.83)	57.88 (51.62, 63.29)	< 0.001
MBP (mmHg)	74.17 (69.42, 79.83)	70.52 (65.92, 76.04)	< 0.001
Resperate (min-1)	20.63 (18.02, 23.30)	22.42 (19.24, 25.46)	< 0.001
Temperature (°C)	36.70 (36.50, 36.97)	36.65 (36.27, 36.91)	< 0.001
**Laboratory tests^b^**			
HB (g/dl)	10.15 (8.70, 11.75)	9.75 (8.50, 11.60)	0.138
HCT (%)	31.05 [26.60, 35.90]	31.10 [26.50, 36.65]	0.627
PLT (109)	148.50 (104.00, 206.50)	159.50 (103.00, 223.50)	0.293
WBC (109)	13.55 (8.45, 19.15)	14.35 (9.65, 19.80)	0.129
L (109)	0.72 (0.44, 1.13)	0.78 (0.44, 1.29)	0.099
M (109)	0.56 (0.30, 0.87)	0.65 (0.34, 1.20)	0.013
N (109)	11.04 (6.82, 17.08)	11.64 (7.39, 17.61)	0.518
ALB (g/dL)	3.00 (2.60, 3.40)	2.70 (2.35, 3.10)	< 0.001
AG (m Eq/L)	15.50 (13.50, 18.00)	20.00 (16.50, 25.00)	< 0.001
BUN (mg/dL)	29.00 (19.00, 42.50)	44.00 (28.00, 64.50)	< 0.001
Cr (μmol/L])	1.35 [0.95, 1.95]	2.05 [1.45, 3.05]	< 0.001
Glucose (mg/dl)	129.50 (105.50, 163.50)	141.50 (108.50, 194.00)	0.001
Sodium (mmo/l)	138.00 (134.50, 140.50)	137.00 (134.00, 141.00)	0.132
Potassium (mmol/l)	4.10 (3.75, 4.55)	4.60 (4.15, 5.10)	< 0.001
Fibrinogen (g/L)	384.50 (252.50, 519.50)	298.00 (195.00, 431.50)	< 0.001
PT (s)	18.65 (16.75, 23.50)	23.15 (17.90, 33.80)	< 0.001
PTT (s)	35.95 (31.05, 44.75)	43.20 (35.20, 60.45)	< 0.001
ALT (U/L)	84.00 (33.00, 199.50)	113.00 (42.50, 402.50)	< 0.001
ALP (U/L)	156.00 (84.50, 288.00)	146.00 (88.50, 273.50)	0.994
AST (U/L)	117.50 (51.00, 228.15)	219.00 (84.00, 807.00)	< 0.001
LDH (U/L)	321.00 (229.50, 528.00)	749.00 (399.00, 1656.00)	<0.001
LAC (mmol/L)	2.65 [1.80, 4.25]	5.00 [3.20, 9.60]	< 0.001
PH	7.40 (7.35, 7.47)	7.36 (7.29, 7.43)	< 0.001
PO2 (mmHg)	153.00 (105.00, 248.00)	154.00 (108.00, 297.00)	0.274
PCO2 (mmHg)	40.00 (34.00, 46.00)	43.00 (34.00, 52.00)	0.006
spo2_mean (%)	96.84 (95.58, 98.12)	95.93 (94.23, 97.63)	< 0.001
Glucose/potassium	31.71 (25.38, 39.47)	30.21 (24.21, 42.63)	0.687
NLR	14.91 (8.14, 27.32)	13.91 (7.44, 25.27)	0.24
PLR	210.01 (120.55, 360.76)	180.93 (102.53, 323.16)	0.049
ALT/ALP	0.47 (0.21, 1.21)	0.74 (0.26, 3.18)	< 0.001
APRI	0.80 (0.36, 1.85)	1.80 (0.59, 5.69)	< 0.001
PNR	14.03 (8.89, 22.50)	13.22 (7.67, 21.69)	0.465
NAR	3.80 (2.15, 5.74)	4.16 (2.53, 6.66)	0.044
SIRI	7.99 (3.37, 18.23)	9.44 (3.12, 23.29)	0.375
**Severity score**			
SAPS II	46.00 (38.00, 55.00)	65.00 (56.00, 74.00)	< 0.001
SIRS	3.00 (2.00, 3.00)	3.00 (3.00, 4.00)	< 0.001
SOFA	4.00 (3.00, 6.00)	5.00 (4.00, 8.00)	< 0.001
GCS	15.00 (14.00, 15.00)	15.00 (13.00, 15.00)	0.845
**Treatment measures**			
CRRT, *n* (%)	23 (3.4)	37 (14.2)	< 0.001
Vasopressor (μg/kg)	1.28 (0.38, 3.48)	3.81 (1.10, 10.26)	< 0.001
Ventilator, *n* (%)	214 (31.8)	171 (65.5)	< 0.001

^a^Vital signs were calculated as mean value during the first 24 h since ICU admission of each included patients.

^b^The laboratory tests recorded the worst value during the first 24 h since ICU admission of each included patients. BMI, body mass index; PLT, blood platelets; L, lymphocyte; M, monocyte; N, neutrophil; SBP, systolic blood pressure; DBP, diastolic blood pressure; MBP, mean arterial pressure; SAPS II, Simplified Acute Physiology Score II; SOFA, Sequential Organ Failure Assessment; SIRS, Systemic Inflammatory Response Syndrome; GCS, Glasgow Coma Scale; HCT, hematocrit; HB, hemoglobin; WBC, white blood cell counts; ALB, albumin; LDH, lactic dehydrogenase; LAC, lactate; AG, anion gap, Cr, creatinine; BUN, blood urine nitrogen; ALT, alanine transaminase; AST, aspartate transaminase; ALP, alkaline phosphatase; PT, prothrombin time; PTT, activated partial thromboplastin time; SpO2, peripheral capillary oxygen saturation; NLR, neutrophil-to-lymphocyte ratio; PLR, platelet-to-lymphocyte ratio; ALT/ALP, alanine transaminase-to-alkaline phosphatase ratio; APRI, aspartate transaminase-to-platelets ratio; PNR, platelet-to-neutrophil ratio; NAR, neutrophil-to-albumin ratio, SIRI, systemic inflammatory response index; CRRT, continuous renal replacement therapy; PUD, peptic ulcer disease; CPD, chronic pulmonary disease; MI, myocardial infarct; RD, renal disease; MC, malignant cancer, DM, diabetes.

### 3.2 Correlation heat map

Correlation matrix and correlation test were performed for all variables for 934 elderly patients and correlation heat map was drawn (Supplementary Figure 2). From the figure, there was much collinearity between various variables, which meant that all the above variables could not be directly included in the logistic regression analysis.

### 3.3 Lasso regression selection of predictor variables

**FIGURE 2:**
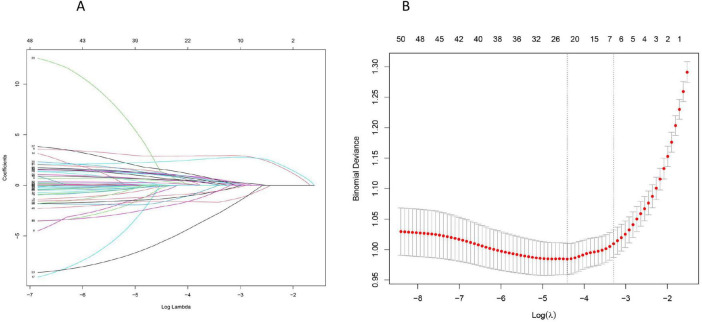
Lasso regression screening of prognostic model predictor variables: **(A)** The selection process of the most appropriate λ in the lasso model; **(B)** Lasso coefficient curves.

According to the correlation heat map, we can conclude that there is collinearity between the variables, which cannot be directly analyzed, so we performed lasso regression to screen out the most suitable variables for establishing the prognosis model of patients. Lasso regression was used to screen the predictor variables of the ML model, and cross-validated lasso fit mean square error (Figure 2A), and lasso fit coefficient trajectory plots (Figure 2B) were used. According to the calculation, when the minimum mean square error of λ is 0.029, the corresponding model predictor variables are selected in detail.

### 3.4 Production of nomograms

**FIGURE 3:**
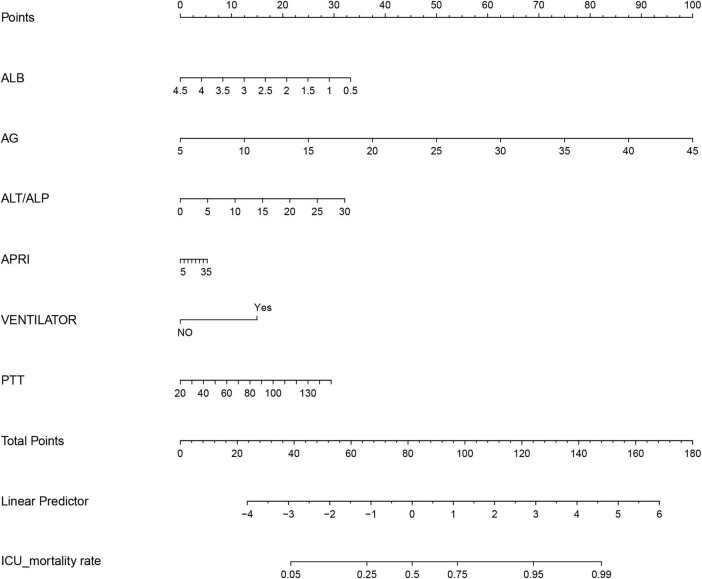
Construction of nomograms.

The data of senile SALI patients were imported into R4.2.2 software, and the data were randomly assigned in a ratio of about 7:3 by setting random seeds. The final number of cases in the training set and the validation set were 653 and 281, respectively (Supplementary Table 2). The variables selected by lasso regression were used to establish a model, final inclusion of albumin (ALB), Anion gap (AG), Activated partial thromboplastin time (PTT), Alanine transaminase-to-Alkaline phosphatase ratio (ALT/ALP), Aspartate transaminase-to-platelets ratio (APRI), and Mechanical ventilation were six variables. According to the “Points” in the nomogram, the individual scores of each variable of a specific individual can be obtained, and the sum of the individual scores can obtain the “Total Points” in the nomogram, and the corresponding “ICU mortality rate” is the ICU mortality probability (Figure 3). To ensure that the variables selected from the Lasso regression conform to the optimal model, we compute the values of Akaike information criterion and Bayesian information criterion, thus validating the statistical model selection and evaluation, as detailed in Supplementary Table 1.

### 3.5 Multivariable logistic analysis

A multifactorial logistic analysis was performed on the six potential predictors (Table 2). The results showed that ALB, AG, PTT, ALT/ALP, APRI, and mechanical ventilation were independent prognostic factors in patients with SALI. To rule out covariance between the variables, we again tested the variables for variance inflation factors, as shown in Supplementary Table 2.

**TABLE 2:** Multivariate logistic analysis.

Variables	OR	95% CI		*P*-value
		**Lower**	**Upper**	
ALB	0.485	0.359	0.655	< 0.001
AG	1.188	1.144	1.234	< 0.000
ALT/ALP	1.070	1.013	1.130	0.015
APRI	1.012	0.968	1.057	0.046
Ventilator	1.013	1.006	1.020	< 0.001
PTT	0.382	0.270	0.539	< 0.001

OR, odds ratio; CI, confidence interval; ALB, albumin; AG, anion gap; ALT/ALP, alanine transaminase-to-alkaline phosphatase ratio; APRI, aspartate transaminase-to-platelets ratio; PTT, activated partial thromboplastin time; PTT, activated partial thromboplastin time.

### 3.6 Discrimination, calibration and evaluation

**FIGURE 4:**
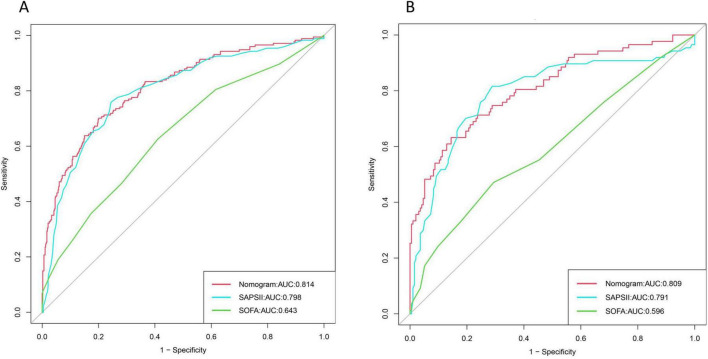
Area under the three curves for nomogram, Simplified Acute Physiology Score II (SAPS II), and Sequential Organ Failure Assessment (SOFA): **(A)** Training set; **(B)** Validation set.

The ROC curve was drawn to evaluate the discrimination of the model. The area under the curve of the training cohort was 0.814 (95% CI: 0.777–0.842), while the area under the curve of the validation cohort was 0.809 (95% CI: 0.752–0.866), which were significantly better than the SAPSII score (AUC of the training cohort: 0.794, 95% CI:0.760–0.828); AUC of the validation cohort: 0.791, 95% CI: 0.727–0.854) and SOFA score (AUC of the training cohort: 0.629, 95% CI: 0.588–0.669; AUC of the validation cohort: 0.596, 95% CI: 0.522–0.670). The specific results are shown in Figure 4.

**FIGURE 5:**
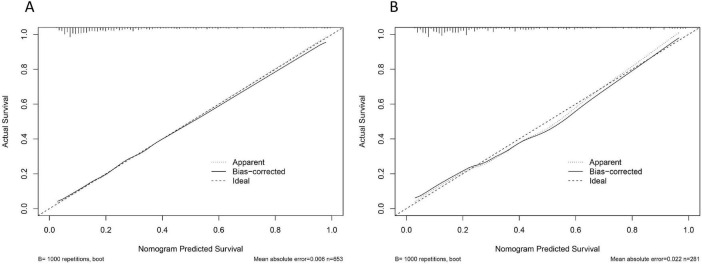
Calibration curve: **(A)** Training set; **(B)** Validation set.

The calibration degree of the model was evaluated by drawing the calibration curve and Hosmer-Lemeshaw goodness of fit test. According to the calibration plot, there was good prediction accuracy between the actual probability and the predicted probability. The *P*-values of the training cohort and validation cohort models are 0.627 and 0.486, respectively, and the fits are good, as shown in Figure 5.

**FIGURE 6:**
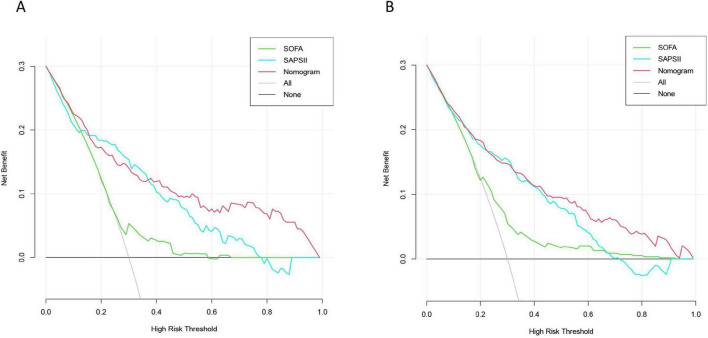
Nomogram, Simplified Acute Physiology Score II (SAPS II), and Sequential Organ Failure Assessment (SOFA) clinical decision curves: **(A)** Training set; **(B)** Validation set.

We use the clinical DCA to evaluate the clinical utility of the model. Within a certain threshold range > 0.4, there are net benefits for both validation and training sets, and the specific results are shown in Figure 6.

The Delong test showed a significant difference in AUROC between our model and the three previous models (*p* < 0.05), indicating that our model performed significantly better than these previous models in predicting ICU mortality in SALI patients. In addition, the results of net reclassification index (NRI) and integrated discrimination index (IDI) showed better reclassification performance of our model. Thus, these results indicate that the predictive accuracy of our model is significantly improved over previous models, as shown in Supplementary Table 3.

## 4 Discussion

Sepsis continues to be a critical concern in modern medicine, demanding urgent attention. If left unchecked, it can escalate into MODS, significantly increasing patient morbidity and mortality (21). Liver failure is an important aspect of septic MODS and usually portends a poor prognosis (22, 23). Despite advances in treatment, mortality rates continue to rise progressively, a problem further exacerbated by the significant aging of today’s population (24). Therefore, it is essential to assess the mortality of elderly SALI patients admitted to the ICU.

This study primarily utilized the MIMIC database to evaluate the incidence and progression of mortality in SALI patients following their admission to the ICU. Lasso regression analysis was employed to identify independent risk factors associated with ICU mortality in these patients. Ultimately, six clinical variables of ALB, AG, PTT, ALT/ALP, APRI and mechanical ventilation were determined by the construction of the model.

Albumin has binding, translocation, and detoxification of endogenous and exogenous molecules, killing effects, antioxidant activity, and modulation of immune and inflammatory responses (25, 26). The mechanisms of SALI are unknown, and previous studies have shown that cytokine-induced inflammation and mitochondrial oxidative stress are key factors contributing to SALI (27). Previous studies have shown that ALB reduces the release of cytochrome c from mitochondria by inhibiting the leakage of the cysteine protease histone B from lysosomes while reducing the release of cytochrome c from mitochondria, concluding that ALB not only protects hepatocytes from the cytotoxic effects of the cytokine TNFα but also prevents inflammatory mediators from further damaging tissues (28). Evidence-based indications for albumin administration in clinical guidelines are primarily liver disease (29). In patients with sepsis or septic shock who remain hemodynamically unstable after resuscitation with 30 ml/kg crystalloid, albumin infusion may be considered clinically for further treatment. It can be added to a large crystalloid infusion rather than used alone for crystalloid therapy (30, 31). In conclusion, when the concentration of ALB in the patient’s body decreases, the mortality rate of the patient will increase, and our modeling further validates that ALB has a strong ability for the prognosis of the patients, and further by strengthening the management of the patient in terms of ALB.

Anion gap is a comprehensive outcome indicator determined by the measured anion and cation concentrations (32). However, AG is also significantly affected by ALB levels (33). In the setting of severe sepsis, the development of hyperlactatemia can trigger a high AG metabolic acidosis. Previous studies have shown that AG in early ICU admission is one of the risk factors for the outcome of sepsis patients (34). In this study, AG variables were included through lasso review to predict the mortality of patients admitted to the ICU, and the effect of ALB on AG was considered, so after screening ALB was also used as a part of the model, which complemented the previous study and thus predicted in several ways.

Alanine transaminase and ALP are sensitive indicators of liver damage, and liver injury can cause elevated serum levels of ALT and ALP (35). ALT is a specific marker of hepatocellular injury, whereas bilirubin and PT-INR represent liver function (36). ALP is an enzyme found primarily in the hepatobiliary tract and is elevated in the cholestatic form of liver injury (37). However, mild to moderate elevations of ALP are common in all types of hepatitis are common, even in hepatocellular injury (36). The ALT/ALP ratio, which combines both ALT and ALP, is more closely related to the pathologic features of liver injury and changes in disease (38). PLT is involved in sepsis combined with multiorgan dysfunction by regulating inflammation, tissue integrity, and defense against infection (39). Studies have shown that APRI is a good predictor of SALI in children that platelets accumulate in the liver when the body is attacked by inflammatory cytokines, leading to liver injury and a decrease in platelet counts in the peripheral blood, and that platelets can influence the regeneration of damaged liver tissue (40). Previous studies have shown that two novel indicators, ALT/ALP and APRI, can be good predictors of SALI, but in the modeling developed in this study, both accounted for a small proportion of the modeling, which may be related to the fact that we did not include enough sample sizes of the elderly. Secondly, ALT and ALP may increase simultaneously in liver injury, resulting in no significant difference in their ratios, and the *P*-value of APRI tends to be close to 0.05 when predicting SALI alone at the beginning of the modeling, which means that it does not have a significant meaning when predicting alone. Therefore, the weighting of the scores in the modeling was not high, which means that we need to include more cases in the future to explore the predictive value of APRI for SALI.

Common complications at the onset of severe sepsis include coagulation abnormalities and liver injury. Overall coagulation indices such as PTT and prothrombin time are generally used in the clinical setting, and a single index may reflect only part of the coagulation system (41). DCIs that occur when there is decreased production of coagulation factors associated with hepatocellular injury or sepsis-associated coagulation abnormalities can exacerbate the depletion. Studies have shown that a longer PTT may reflect the most severe sepsis due to coagulation factor depletion or high-dose heparin therapy and that a prolonged PTT is associated with higher mortality (42), and PTT prolongation is also an important marker of severe hyperfibrinolysis (43). This is consistent with our study. As the PTT value prolongs, the percentage of PTT increases, leading to a gradual rise in patient mortality rates. The occurrence of SALI further impacts the consumption of coagulation factors, resulting in sepsis-related coagulation abnormalities. In severe cases, DIC develops, accelerating patient mortality. These two conditions exacerbate each other, so it is crucial to clinically monitor changes in coagulation function when SALI occurs.

The last variable incorporated in our model is mechanical ventilation. When a patient is in a critical situation, such as respiratory failure, respiratory arrest, or oxygen desaturation, clinical work is mostly done with a ventilator. Mechanical ventilation is widely used in the management of septic patients but is prone to complications. Ventilator-associated pneumonia, a lung infection during mechanical ventilation, is the most common nosocomial lung infection in intensive care patients. Whereas sepsis itself, as a severe systemic inflammatory disease, its combination with ventilator-associated pneumonia may lead to deterioration of the patient’s condition and increase the risk of death (44, 45).

Simplified Acute Physiology Score II and SOFA scores are commonly used to evaluate the prognosis of patients in clinical practice (5, 46), so the two variables are not included in the model construction. In this study, SOFA and SAPS2 scores were used to compare their performance with that of predictive models. We found that nomogram performed the best. In addition, the DCA curve supports this finding. Through the above comparison, it is further shown that the efficiency of the constructed model is better.

Intensive care unit mortality in elderly SALI patients was the endpoint of this study. Independent risk factors were screened by lasso regression, and a risk prediction model was successfully constructed based on these factors. Lasso regression analysis can solve the problem of multicollinearity among variables and is superior to univariate analysis. The nomogram constructed based on the Lasso regression model has a certain reference value for medical workers to visually analyze individual prognosis. As a visual scoring tool suitable for clinical research, the nomogram can synthesize various influencing factors and present their results intuitively. Decision makers can formulate individualized treatment plans to prolong survival based on the predicted risk of death. Through the predictive model, doctors can find out in advance whether a patient is likely to have a poor prognosis and take appropriate measures to reduce the risk of mortality, thus improving treatment outcomes, lowering healthcare costs, and enhancing patients’ quality of life.

## 5 Strengths and limitation

By establishing a robust predictive model, clinicians can proactively identify health issues at an early stage, thereby improving both treatment efficacy and the overall quality of life for patients. However, it is crucial to recognize the limitations inherent in this study. Firstly, it relies on a single-center retrospective analysis, and the findings require validation through prospective cohort studies. Secondly, despite the approach of randomly dividing the data into training and validation sets, and the highly reliable models produced during the development process, the generalizability of the models is still limited by the lack of external validation of the centralized dataset. This limitation emphasizes the need for further evaluation of its stability and applicability. Lastly, while this analysis focused on 934 elderly patients, future research would benefit from multi-center approaches and larger-scale validations to enhance both stability and performance.

## 6 Conclusion

We successfully constructed a model of in-ICU mortality of SALI elderly patients. The nomogram created based on the above factors assessed the risk of death in patients, which is of great informative value and clinical applicability.

## Data Availability

The original contributions presented in this study are included in this article/Supplementary material, further inquiries can be directed to the corresponding author.

## References

[1] Singer M Deutschman CS Seymour CW Shankar-Hari M Annane D Bauer M The third international consensus definitions for sepsis and septic shock (Sepsis-3). *JAMA.* (2016) 315:801–10. 10.1001/jama.2016.0287 26903338 PMC4968574

[2] Yan J Li S Li S. The role of the liver in sepsis. *Int Rev Immunol.* (2014) 33:498–510. 10.3109/08830185.2014.889129 24611785 PMC4160418

[3] Dai JM Guo WN Tan YZ Niu KW Zhang JJ Liu CL Wogonin alleviates liver injury in sepsis through Nrf2-mediated NF-κB signalling suppression. *J Cell Mol Med.* (2021) 25:5782–98. 10.1111/jcmm.16604 33982381 PMC8184690

[4] Wang J Zhu Q Li R Zhang J Ye X Li X. YAP1 protects against septic liver injury via ferroptosis resistance. *Cell Biosci.* (2022) 12:163. 10.1186/s13578-022-00902-7 36182901 PMC9526934

[5] Hu T Lv H Jiang Y. The association between four scoring systems and 30-day mortality among intensive care patients with sepsis: A cohort study. *Sci Rep.* (2021) 11:11214. 10.1038/s41598-021-90806-2 34045645 PMC8159970

[6] Dhainaut JF Marin N Mignon A Vinsonneau C. Hepatic response to sepsis: Interaction between coagulation and inflammatory processes. *Crit Care Med.* (2001) 29:S42–7. 10.1097/00003246-200107001-00016 11445733

[7] Sun J Zhang J Wang X Ji F Ronco C Tian J Gut-liver crosstalk in sepsis-induced liver injury. *Crit Care.* (2020) 24:614. 10.1186/s13054-020-03327-1 33076940 PMC7574296

[8] Kobashi H Toshimori J Yamamoto K. Sepsis-associated liver injury: Incidence, classification and the clinical significance. *Hepatol Res.* (2013) 43:255–66. 10.1111/j.1872-034X.2012.01069.x 22971102

[9] Henrion J Schapira M Luwaert R Colin L Delannoy A Heller FR. Hypoxic hepatitis: Clinical and hemodynamic study in 142 consecutive cases. *Medicine (Baltimore).* (2003) 82:392–406. 10.1097/01.md.0000101573.54295.bd 14663289

[10] Bhogal HK Sanyal AJ. The molecular pathogenesis of cholestasis in sepsis. *Front Biosci (Elite Ed).* (2013) 5:87–96. 10.2741/e598 23276972 PMC4944118

[11] Fontana RJ Seeff LB Andrade RJ Björnsson E Day CP Serrano J Standardization of nomenclature and causality assessment in drug-induced liver injury: Summary of a clinical research workshop. *Hepatology.* (2010) 52:730–42. 10.1002/hep.23696 20564754 PMC3616501

[12] Zhang W Jiang H Wu G Huang P Wang H An H The pathogenesis and potential therapeutic targets in sepsis. *MedComm (2020).* (2023) 4:e418. 10.1002/mco2.418 38020710 PMC10661353

[13] Ji Y Yin Y Li Z Zhang W. Gut microbiota-derived components and metabolites in the progression of non-alcoholic fatty liver disease (NAFLD). *Nutrients.* (2019) 11:1712. 10.3390/nu11081712 31349604 PMC6724003

[14] Lv M Wang C Li F Peng J Wen B Gong Q Structural insights into the recognition of phosphorylated FUNDC1 by LC3B in mitophagy. *Protein Cell.* (2017) 8:25–38. 10.1007/s13238-016-0328-8 27757847 PMC5233613

[15] Vanhorebeek I Gunst J Derde S Derese I Boussemaere M D’Hoore A Mitochondrial fusion, fission, and biogenesis in prolonged critically ill patients. *J Clin Endocrinol Metab.* (2012) 97:E59–64. 10.1210/jc.2011-1760 22013100

[16] Woźnica EA Inglot M Woźnica RK Łysenko L. Liver dysfunction in sepsis. *Adv Clin Exp Med.* (2018) 27:547–51. 10.17219/acem/68363 29558045

[17] Iwashyna TJ Cooke CR Wunsch H Kahn JM. Population burden of long-term survivorship after severe sepsis in older Americans. *J Am Geriatr Soc.* (2012) 60:1070–7. 10.1111/j.1532-5415.2012.03989.x 22642542 PMC3374893

[18] Jalali A Alvarez-Iglesias A Roshan D Newell J. Visualising statistical models using dynamic nomograms. *PLoS One.* (2019) 14:e0225253. 10.1371/journal.pone.0225253 31730633 PMC6857916

[19] Johnson AEW Bulgarelli L Shen L Gayles A Shammout A Horng S MIMIC-IV, a freely accessible electronic health record dataset. *Sci Data.* (2023) 10:1. 10.1038/s41597-022-01899-x 36596836 PMC9810617

[20] Pruinelli L Westra BL Yadav P Hoff A Steinbach M Kumar V Delay within the 3-hour surviving sepsis campaign guideline on mortality for patients with severe sepsis and septic shock. *Crit Care Med.* (2018) 46:500–5. 10.1097/ccm.0000000000002949 29298189 PMC5851815

[21] Hotchkiss RS Moldawer LL Opal SM Reinhart K Turnbull IR Vincent JL. Sepsis and septic shock. *Nat Rev Dis Primers.* (2016) 2:16045. 10.1038/nrdp.2016.45 28117397 PMC5538252

[22] Beyer D Hoff J Sommerfeld O Zipprich A Gaßler N Press AT. The liver in sepsis: Molecular mechanism of liver failure and their potential for clinical translation. *Mol Med.* (2022) 28:84. 10.1186/s10020-022-00510-8 35907792 PMC9338540

[23] Tanaka S De Tymowski C Stern J Bouzid D Zappella N Snauwaert A Relationship between liver dysfunction, lipoprotein concentration and mortality during sepsis. *PLoS One.* (2022) 17:e0272352. 10.1371/journal.pone.0272352 35994439 PMC9394828

[24] Eshghi F Tahmasebi S Alimohammadi M Soudi S Khaligh SG Khosrojerdi A Study of immunomodulatory effects of mesenchymal stem cell-derived exosomes in a mouse model of LPS induced systemic inflammation. *Life Sci.* (2022) 310:120938. 10.1016/j.lfs.2022.120938 36150466

[25] Fanali G di Masi A Trezza V Marino M Fasano M Ascenzi P. Human serum albumin: From bench to bedside. *Mol Aspects Med.* (2012) 33:209–90. 10.1016/j.mam.2011.12.002 22230555

[26] Casulleras M Flores-Costa R Duran-Güell M Alcaraz-Quiles J Sanz S Titos E Albumin internalizes and inhibits endosomal TLR signaling in leukocytes from patients with decompensated cirrhosis. *Sci Transl Med.* (2020) 12:aax5135. 10.1126/scitranslmed.aax5135 33087502

[27] Duran-Güell M Garrabou G Flores-Costa R Casulleras M López-Vicario C Zhang IW Essential role for albumin in preserving liver cells from TNFα-induced mitochondrial injury. *Faseb J.* (2023) 37:e22817. 10.1096/fj.202201526R 36809676

[28] Duran-Güell M Flores-Costa R Casulleras M López-Vicario C Titos E Díaz A Albumin protects the liver from tumor necrosis factor α-induced immunopathology. *Faseb J.* (2021) 35:e21365. 10.1096/fj.202001615RRR 33496031

[29] Abedi F Zarei B Elyasi S. Albumin: A comprehensive review and practical guideline for clinical use. *Eur J Clin Pharmacol.* (2024) 80:1151–69. 10.1007/s00228-024-03664-y 38607390

[30] Yu SH Ma YT Li X. [The correlation between coagulation function and prognosis in patients with acute respiratory distress syndrome caused by extrapulmonary sepsis or pulmonary infection]. *Zhonghua Nei Ke Za Zhi.* (2021) 60:650–5. 10.3760/cma.j.cn112138-20201217-01017 34619843

[31] Evans L Rhodes A Alhazzani W Antonelli M Coopersmith CM French C Surviving sepsis campaign: International guidelines for management of sepsis and septic shock 2021. *Crit Care Med.* (2021) 49:e1063–143. 10.1097/ccm.0000000000005337 34605781

[32] Oh MS Carroll HJ. The anion gap. *N Engl J Med.* (1977) 297:814–7. 10.1056/nejm197710132971507 895822

[33] Nanji AA Campbell DJ Pudek MR. Decreased anion gap associated with hypoalbuminemia and polyclonal gammopathy. *JAMA.* (1981) 246:859–60. 6166764

[34] Lou Z Zeng F Huang W Xiao L Zou K Zhou H. Association between the anion-gap and 28-day mortality in critically ill adult patients with sepsis: A retrospective cohort study. *Medicine (Baltimore).* (2024) 103:e39029. 10.1097/md.0000000000039029 39058855 PMC11272324

[35] Meng YJ Yu HL Yang S. Clinical significance of GLDH, GGT, ALT and ALP combined detection in the diagnosis of drug-induced liver injury. *Lab Med Clinic.* (2019) 16:1735–7. 10.3969/j.issn.1672-9455.2019.12.032

[36] Kwo PY Cohen SM Lim JK. ACG clinical guideline: Evaluation of abnormal liver chemistries. *Am J Gastroenterol.* (2017) 112:18–35. 10.1038/ajg.2016.517 27995906

[37] Kalas MA Chavez L Leon M Taweesedt PT Surani S. Abnormal liver enzymes: A review for clinicians. *World J Hepatol.* (2021) 13:1688–98. 10.4254/wjh.v13.i11.1688 34904038 PMC8637680

[38] Chunyang H Zhang XD Huang YL Han Y Li WJ Serum ALT/ALP ratio changes and histopathological features of patients with drug-induced liver injury acute hepatocellular type. *J Pract Hepatol.* (2021) 24:379–82. 10.3969/j.issn.1672-5069.2021.03.019

[39] Graham SM Liles WC. Platelets in sepsis: Beyond hemostasis. *Blood.* (2016) 127:2947–9. 10.1182/blood-2016-03-706168 27313324

[40] Dou J Zhou Y Cui Y Chen M Wang C Zhang Y. AST-to-platelet ratio index as potential early-warning biomarker for sepsis-associated liver injury in children: A database study. *Front Pediatr.* (2019) 7:331. 10.3389/fped.2019.00331 31497584 PMC6713043

[41] Mohapatra P Kumar A Singh RK Gupta R Hussain M Singh S The effect of sepsis and septic shock on the viscoelastic properties of clot quality and mass using thromboelastometry: A prospective observational study. *Indian J Crit Care Med.* (2023) 27:625–34. 10.5005/jp-journals-10071-24539 37719352 PMC10504658

[42] Niederwanger C Bachler M Hell T Linhart C Entenmann A Balog A Inflammatory and coagulatory parameters linked to survival in critically ill children with sepsis. *Ann Intensive Care.* (2018) 8:111. 10.1186/s13613-018-0457-8 30446841 PMC6240023

[43] Martin-Loeches I. Current concepts in community and ventilator associated lower respiratory tract infections in ICU patients. *Antibiotics (Basel).* (2020) 9:380. 10.3390/antibiotics9070380 32635601 PMC7399936

[44] Gopalakrishnan R Vashisht R. Sepsis and ECMO. *Indian J Thorac Cardiovasc Surg.* (2021) 37:267–74. 10.1007/s12055-020-00944-x 32421057 PMC7223121

[45] Lambden S Laterre PF Levy MM Francois B. The SOFA score-development, utility and challenges of accurate assessment in clinical trials. *Crit Care.* (2019) 23:374. 10.1186/s13054-019-2663-7 31775846 PMC6880479

[46] Yu YT Liu J Hu B Wang RL Yang XH Shang XL Expert consensus on the use of human serum albumin in critically ill patients. *Chin Med J (Engl).* (2021) 134:1639–54. 10.1097/cm9.0000000000001661 34397592 PMC8318641

