# Supplementary figures and images for: Development of a nomogram to predict in-ICU mortality of elderly patients with sepsis-associated liver injury: an analysis of the MIMIC-IV database
Source: Front Med (Lausanne). 2025 Mar 26;12:1516853. doi: 10.3389/fmed.2025.1516853 (PMC11979112; doi:10.3389/fmed.2025.1516853)

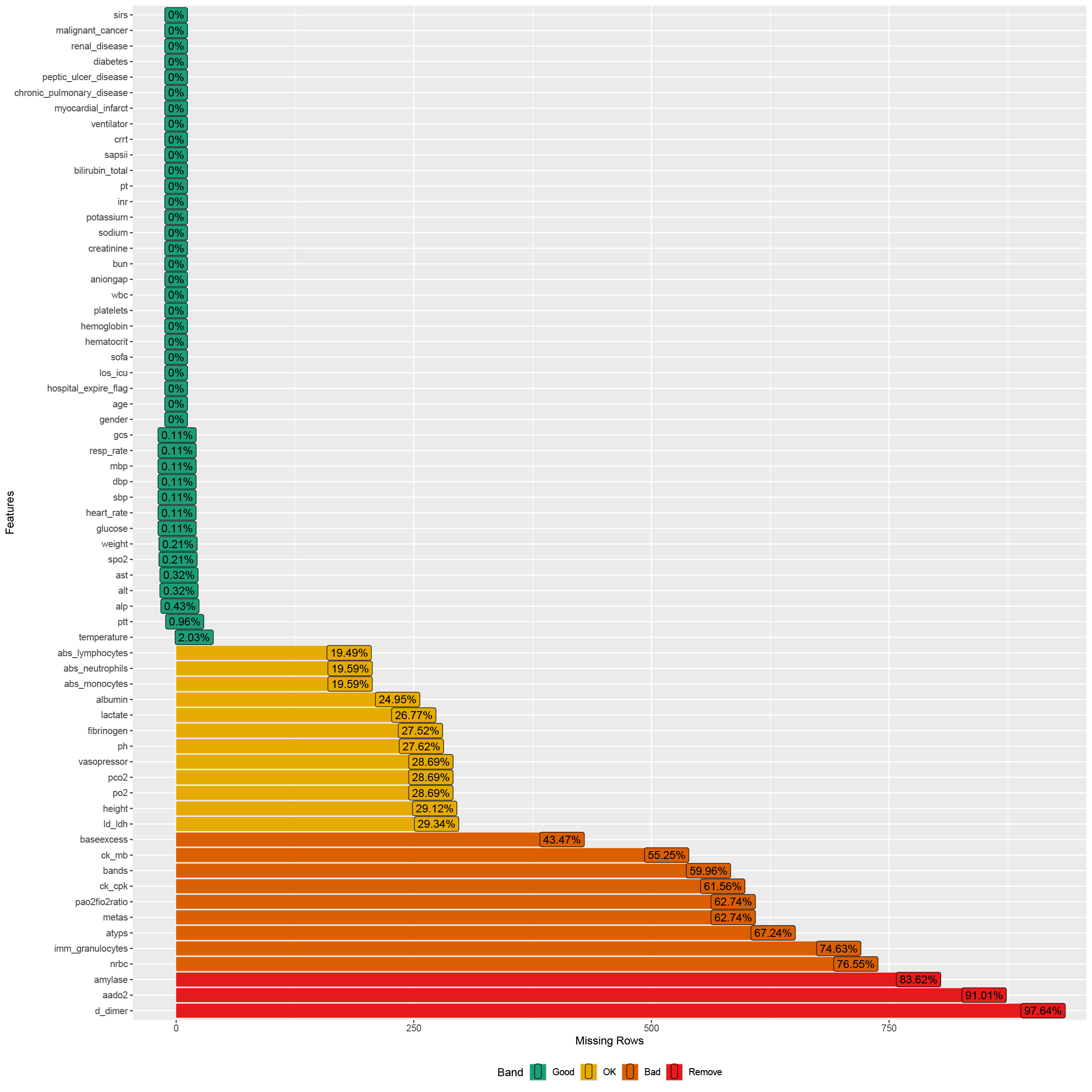

Supplement: Supplementary file 1 [file Image_1.TIF]

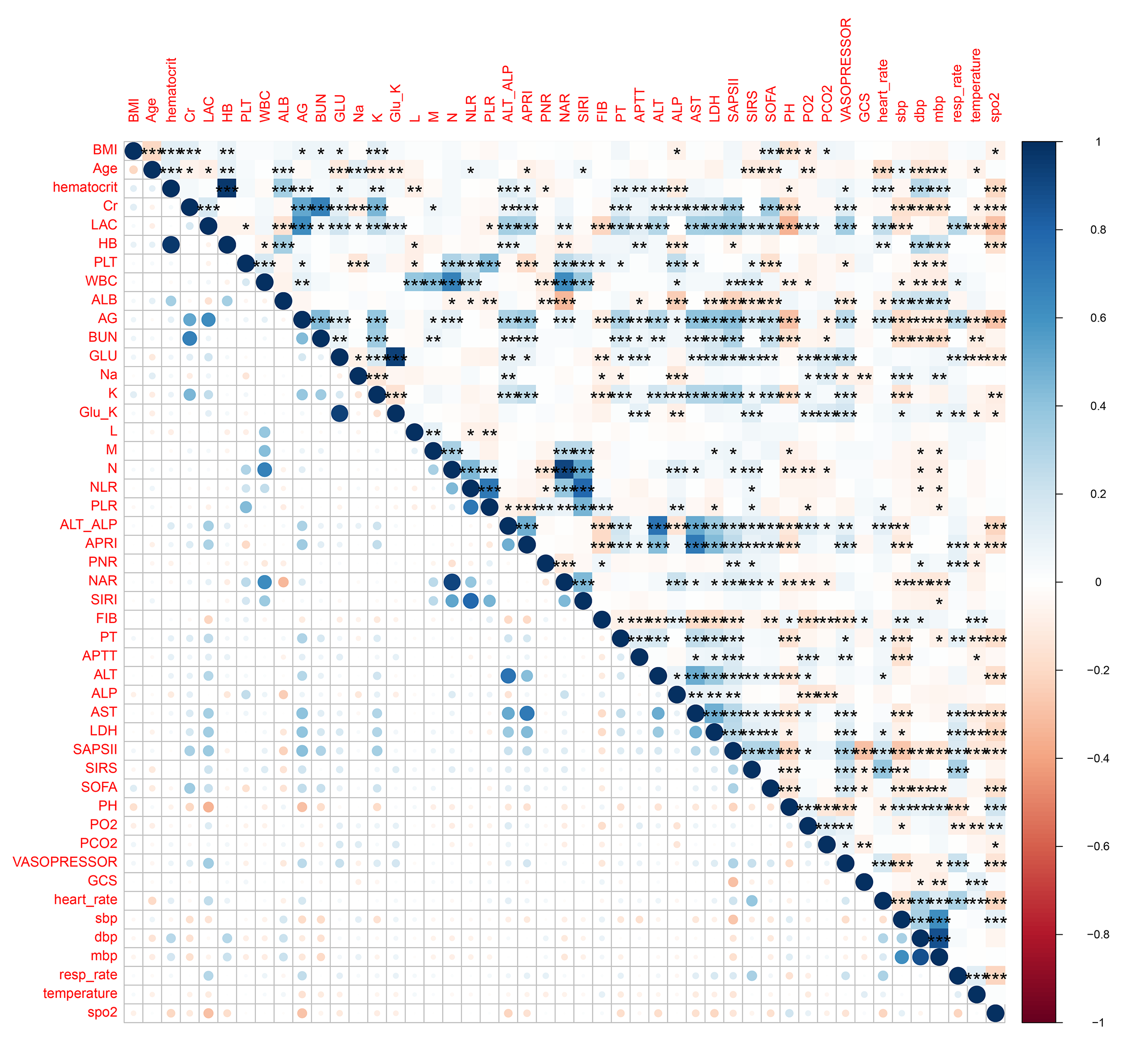

Supplement: Supplementary file 2 [file Image_2.TIF]
