# Supplementary material for: Development of a nomogram to predict in-ICU mortality of elderly patients with sepsis-associated liver injury: an analysis of the MIMIC-IV database
Source: Front Med (Lausanne). 2025 Mar 26;12:1516853. doi: 10.3389/fmed.2025.1516853 (PMC11979112; doi:10.3389/fmed.2025.1516853)
Supplement: Supplementary file 3 [file Table_1.DOCX]

**supplementary table 1** Installation packages used for analyzing data usage

| Installation packages | explanatory notes (Figure or Table) |
| --- | --- |
| " CBCgrps" and "tableone" | Table 1 and supplementary table 2 |
| "glmnet" | Lasso regression (Figure 2) |
| "Rms" | Nomogram (Figure 3) |
| "pROC" and "ggplot2" | ROC (Figure 4) |
| "caret " | Calibration curves (Figure 5) |
| "rmda" | Decision curve analysis (Figure 6) |
| "DataExplorer" | Calculation and treatment of missing values (Supplementary Figure 1) |
| "corrplot" | correlation heat map (Supplementary Figure 2) |
