# Supplementary material for: Development of a nomogram to predict in-ICU mortality of elderly patients with sepsis-associated liver injury: an analysis of the MIMIC-IV database
Source: Front Med (Lausanne). 2025 Mar 26;12:1516853. doi: 10.3389/fmed.2025.1516853 (PMC11979112; doi:10.3389/fmed.2025.1516853)
Supplement: Supplementary file 4 [file Table_2.DOCX]

Supplementary Table 1 Baseline Characteristics of Patients in the training and validation set

| **Variables** | **training set(n=653)** | **validation set(n=281)** | ***P*-value** |
| --- | --- | --- | --- |
| **General features** |  |  |  |
| gender, woman (%) | 403 (61.7) | 166 (59.1) | 0.493 |
| BMI (kg/m2) | 27.62 (23.67, 31.81) | 27.43 (23.85, 31.47) | 0.976 |
| Age (year) | 75.85 (69.05, 83.09) | 76.41 (69.54, 83.98) | 0.512 |
| **Comorbidity, n (%)** |  |  |  |
| MI, n (%) | 143 (21.9) | 60 (21.4) | 0.921 |
| CPD, n (%) | 147 (22.5) | 79 (28.1) | 0.08 |
| PUD, (%) | 26 (4.0) | 14 (5.0) | 0.606 |
| DM, n (%) | 181 (27.7) | 78 (27.8) | 1 |
| RD, n (%) | 209 (32.0) | 89 (31.7) | 0.981 |
| MC n (%) | 157 (24.0) | 69 (24.6) | 0.933 |
| **Vital signs a** |  |  |  |
| heartrate (min-1) | 88.00 (77.82, 101.96) | 90.64 (77.60, 103.15) | 0.458 |
| SBP (mmHg) | 107.63 (99.87, 116.46) | 108.11 (101.34, 116.67) | 0.279 |
| DBP ((mmHg)) | 59.18 (53.49, 64.65) | 59.31 (54.04, 67.08) | 0.288 |
| MBP ((mmHg)) | 73.15 (68.13, 78.14) | 73.26 (68.25, 80.00) | 0.423 |
| resprate (min-1) | 21.04 (18.19, 23.88) | 21.04 (18.57, 23.96) | 0.513 |
| temperature(℃) | 36.69 (36.49, 36.95) | 36.70 (36.49, 36.93) | 0.99 |
| **Laboratory tests b** |  |  |  |
| HB (g/dl) | 9.95 (8.60, 11.65) | 10.25 (8.75, 11.90) | 0.149 |
| HCT(%) | 30.70 (26.55, 35.80) | 31.65 (26.50, 36.60) | 0.093 |
| PLT (109) | 153.50 (104.00, 212.00) | 152.50 (102.50, 213.00) | 0.973 |
| WBC (109) | 13.85 (8.75, 19.55) | 13.70 (9.15, 19.35) | 0.837 |
| L(109) | 0.72 (0.42, 1.14) | 0.78 (0.48, 1.19) | 0.257 |
| M(109) | 0.56 (0.29, 0.93) | 0.62 (0.36, 0.98) | 0.067 |
| N (109) | 11.21 (6.83, 17.52) | 11.04 (7.15, 16.47) | 0.694 |
| ALB (g/dL) | 2.90 (2.50, 3.30) | 2.95 (2.50, 3.35) | 0.293 |
| AG (m Eq/L) | 16.50 (14.00, 20.00) | 16.50 (14.00, 19.00) | 0.623 |
| BUN (mg/dL) | 31.00 (21.00, 48.50) | 33.00 (20.50, 50.00) | 0.797 |
| Cr (umol/L)) | 1.50 (1.05, 2.35) | 1.50 (1.00, 2.25) | 0.256 |
| glucose (mg/dl) | 131.50 (107.00, 170.00) | 130.00 (103.00, 176.50) | 0.663 |
| Sodium (mmo/l) | 137.50 (134.50, 141.00) | 138.00 (134.00, 140.50) | 0.717 |
| Potassium (mmol/l) | 4.25 (3.85, 4.70) | 4.20 (3.80, 4.75) | 0.68 |
| Fibrinogen (g/L) | 350.50 (218.00, 491.00) | 378.00 (253.00, 516.00) | 0.057 |
| PT (s) | 19.35 (16.95, 25.00) | 19.70 (17.10, 28.10) | 0.298 |
| PTT (s) | 37.10 (32.00, 49.50) | 37.20 (31.85, 47.90) | 0.569 |
| ALT (U/L) | 94.00 (38.00, 240.50) | 87.00 (31.50, 213.50) | 0.177 |
| ALP (U/L) | 156.00 (88.00, 300.00) | 152.50 (84.00, 240.00) | 0.186 |
| AST (U/L) | 139.50 (65.00, 326.00) | 117.50 (55.00, 262.00) | 0.022 |
| LDH (U/L) | 375.50 (253.00, 800.00) | 374.00 (249.50, 722.00) | 0.63 |
| LAC (mmol/L) | 3.35 (2.00, 5.30) | 2.65 (1.85, 5.00) | 0.01 |
| PH | 7.39 (7.33, 7.46) | 7.40 (7.33, 7.45) | 0.86 |
| PO2 (mmHg) | 154.00 (106.00, 265.00) | 150.00 (105.00, 246.00) | 0.427 |
| PCO2 (mmHg) | 40.00 (34.00, 49.00) | 40.00 (34.00, 48.00) | 0.757 |
| spo2_mean (%) | 96.68 (95.26, 98.00) | 96.45 (95.20, 97.84) | 0.321 |
| glucose/Potassium | 31.07 (25.19, 39.70) | 31.49 (24.30, 41.23) | 0.912 |
| NLR | 14.89 (7.75, 27.28) | 14.03 (8.15, 25.27) | 0.336 |
| PLR | 207.88 (114.85, 352.78) | 190.58 (115.53, 323.16) | 0.313 |
| ALT/ALP | 0.52 (0.21, 1.43) | 0.51 (0.23, 1.30) | 0.985 |
| APRI | 0.98 (0.44, 2.87) | 0.88 (0.34, 2.37) | 0.122 |
| PNR | 13.69 (8.69, 21.86) | 14.51 (8.21, 22.42) | 0.947 |
| NAR | 3.90 (2.28, 6.23) | 3.83 (2.16, 5.70) | 0.492 |
| SIRI | 8.06 (3.15, 19.03) | 8.72 (3.85, 18.80) | 0.667 |
| **Severity score** |  |  |  |
| SAPSII | 51.00 (40.00, 64.00) | 50.00 (41.00, 60.00) | 0.602 |
| SIRS | 3.00 (2.00, 4.00) | 3.00 (3.00, 4.00) | 0.146 |
| SOFA | 4.00 (3.00, 6.00) | 4.00 (3.00, 6.00) | 0.087 |
| GCS | 15.00 (14.00, 15.00) | 15.00 (14.00, 15.00) | 0.661 |
| **Treatment measures** |  |  |  |
| CRRT, n (%) | 40 (6.1) | 20 (7.1) | 0.673 |
| Vasopressor (ug/kg) | 1.81 (0.55, 4.99) | 1.40 (0.40, 4.79) | 0.237 |
| Ventilator, n(%) | 269 (41.2) | 116 (41.3) | 1 |

Note: a Vital signs were calculated as mean value during the first 24 h since ICU admission of each included patients.

b The laboratory tests recorded the worest value during the first 24 h since ICU admission of each included patients.

BMI Body mass index, PLT Blood platelets, L lymphocyte, M monocyte, N neutrophil, SBP systolic blood pressure, DBP Diastolic blood pressure, MBP mean arterial pressure, SAPS II Simplifed Acute Physiology Score II, SOFA Sequential Organ Failure Assessment, SIRS Systemic Inflammatory Response Syndrome, GCS Glasgow Coma Scale, HCT hematocrit, HB hemoglobin, WBC white blood cell counts, ALB albumin, LDH lactic dehydrogenase, LAC Lactate, AG Anion gap, Cr creatinine, BUN blood urine nitrogen, ALT Alanine transaminase, AST Aspartate Transaminase, ALP Alkaline phosphatase, PT Prothrombin time, PTT Activated partial thromboplastin time, SpO2 Peripheral capillary oxygen saturation, NLR Neutrophil-to-lymphocyte ratio, PLR Platelet-to-lymphocyte ratio, ALT/ALP Alanine transaminase-to-Alkaline phosphatase ratio, APRI Aspartate Transaminase-to-platelets ratio, PNR Platelet-to-Neutrophil ratio, NAR Neutrophil-to-Albumin ratio, SIRI Systemic inflammatory response index, CRRT Continuous renal replacement therapy, PUD peptic ulcer disease , CPD Chronic pulmonary disease, MI Myocardial infarct, RD renal disease, MC malignant cancer, DM diabetes
