# Supplementary material for: Development of a nomogram to predict in-ICU mortality of elderly patients with sepsis-associated liver injury: an analysis of the MIMIC-IV database
Source: Front Med (Lausanne). 2025 Mar 26;12:1516853. doi: 10.3389/fmed.2025.1516853 (PMC11979112; doi:10.3389/fmed.2025.1516853)
Supplement: Supplementary file 5 [file Table_3.DOCX]

Supplementary TableS1 Assessment of the goodness-of-ft of models.

|  | Nomogram | SAPSII | SOFA |
| --- | --- | --- | --- |
| AIC | 873.3924 | 937.9128 | 1053.636 |
| BIC | 883.0713 | 971.7892 | 1063.315 |
| p-value | ＜0.001 | ＜0.001 | ＜0.001 |

Note: AIC Akaike information criterion, BIC Bayesian information criterion.

Supplementary TableS2 Variance inflation factor of each variable in the model.

| Variables | Variance inflation factor |
| --- | --- |
| ALB | 1.001 |
| AG | 1.216 |
| ALT/ALP | 1.007 |
| APRI | 1.019 |
| Ventilator | 1.439 |
| PTT | 1.587 |

Supplementary TableS3 Performance metrics.

|  | Nomogram | p-value | SAPSII | p-value | SOFA | p-value |
| --- | --- | --- | --- | --- | --- | --- |
| AUROC | 0.814 | ＜0.001^#^ | 0.794 | 0.001^#^ | 0.629 | ＜0.001^#^ |
|  | 0.777-0.842 |  | 0.760-0.828 |  | 0.588–0.669 |  |
| NRI | 0.8486 | ＜0.001 | 0.6101 | ＜0.001 | 0.2828 | ＜0.001 |
|  | 0.7179-0.9794 |  | 0.4731-0.7471 |  | 0.1410-0.4245 |  |
| IDI | 0.1938 | ＜0.001 | 0.1351 | ＜0.001 | 0.0586 | ＜0.001 |
|  | 0.1604-0.2272 |  | 0.1015-0.1688 |  | 0.0214-0.0959 |  |

AUROC: area under the receiver operating characteristic curve; NRI: net reclassification index; IDI: integrated discrimination index; #: results of Delong’ test compared to our model.
